# Factors Affecting Survival in Nontraumatic Pediatric Abdominal Surgical Emergencies: A Contemporary Review

**DOI:** 10.7759/cureus.24891

**Published:** 2022-05-10

**Authors:** Harshal Tayade, Yashwant Lamture, Meenakshi Yeola

**Affiliations:** 1 Department of General Surgery, Jawaharlal Nehru Medical College, Datta Meghe Institute of Medical Sciences (Deemed to be University), Wardha, IND; 2 Department of General Surgery, All India Institute of Medical Sciences, Mangalagiri, IND

**Keywords:** pediatric nontraumatic surgical emergency of the abdomen, pediatric abdominal surgical emergency, acute abdominal surgical emergency in children, abdominal surgical emergency in children, acute abdomen in children

## Abstract

Surgically curable illnesses in the pediatric population are a major public health issue with a high prevalence of 10%-33% of all pediatric admissions, and emergency situations account for 50%-78% of surgical cases. Emergency abdominal surgery in children necessitates proper and prompt surgical and perioperative supportive care. When compared to elective operations, emergency surgery has a greater rate of morbidity and fatality. Staffing concerns, access to operating theaters, and access to diagnostic investigations are all possible causes of this high fatality rate, in addition to patient-related factors. Literature from high-income countries (HICs) discusses the problem, and recommendations are available for high-quality setups with good infrastructure. However, surgical care facilities from resource-poor countries have altogether different challenges and bottlenecks when dealing with children requiring emergency surgical operative procedures to save lives. This review aims to discuss factors affecting the survival of children being operated on for abdominal emergencies in resource-poor setups and suggest recommendations.

## Introduction and background

In low- and middle-income countries (LMICs), surgically curable illnesses in the pediatric population are a major public health issue [1], and the scale of surgically preventable disease burden exceeds that of some of the world's most widely discussed health issues. The prevalence of abdominal pain among the pediatric age group reporting to the emergency department varies around the world. Surgical conditions account for 10%-33% of all pediatric admissions, with emergency situations accounting for 50%-78% of surgical cases [2]. In children presenting to the emergency department with acute abdominal pain, the incidence of acute surgical emergency ranges from 10% to 30%; however, in general, the incidence of surgical acute abdominal pain is 2% [3]. According to worldwide sickness burden estimates, surgical illnesses account for 6%-12% of all pediatric admissions in low- and middle-income countries. Emergency surgical conditions in children, unlike elective surgery or the same pathology in adults, necessitate proper and prompt surgical and perioperative supportive care [2]. A multidisciplinary team approach is required for its management. Qualified surgeons, nurses, anesthetists, and caregivers form the pediatric surgery team, which provides intensive care to children. Available literature from high-income countries discusses the determinants of survival in high-quality setups [3-5]. In studies from low- and middle-income countries (LMICs), sepsis, multiple procedures, postoperative hemorrhage, and complicated congenital anomalies have all been linked to perioperative mortality. Lack of skilled personnel, delayed presentation, birthing outside of a hospital, and financial limitations of caregivers are all risk factors. When compared to elective operations, emergency surgery has a greater rate of morbidity and mortality. Staffing concerns, access to operating theaters, and access to diagnostic investigations are all possible causes of this high fatality rate, in addition to patient-related factors [6].

This review aims at discussing the various factors affecting the survival of children undergoing operative procedures for nontraumatic abdominal emergencies in various setups and drawing recommendations for practice.

## Review

Methods

We searched the PubMed database, MEDLINE, EMBASE, ISI Web of Science, and Google Scholar using the MeSH terms pediatric, abdominal, surgical, and emergencies and found 1,418 articles from 1958 to 2021. When we restricted our search from 2001 to 2021, 30 relevant articles could be accessed, of which a maximum of 20 are from the South African region and five are from India. The sample size ranged from a minimum of 68 to a maximum of 849,445, with an age range from 0 to 21 years. The articles included in the review are shown in Table 1.

**Table 1:** Relevant articles included in the review

Author	Year	Place	Sample size	Age group	Perioperative mortality
Ameh et al. [7]	2001	Zaria, Nigeria	154	0-28 days	30.50%
Keita et al. [8]	2006	Donka, Republic of Guinea	222	0-28 days	29.28%
Mhando et al. [9]	2007	Tanzania	110	0-14 years	24%
Tseng et al. [10]	2007	Changhua, Taiwan	400	0-16 years	0.04%
Osifo et al. [11]	2008	Benin City, Nigeria	118	12 hours-28 days	30.5%
Abantanga et al. [12]	2009	Kumasi, Ghana	955	0-14 years	9.7%
Ekenze et al. [13]	2010	Enugu, Nigeria	115	0-14 years	8.70%
Ghritlaharey et al. [14]	2011	Bhopal, India	334	0-12 years	10.17%
Ademuyiwa et al. [15]	2012	Lagos, Nigeria	129	0-15 years	10.07%
Olajide et al. [16]	2012	Ilorin	100	0-12 years	11%
Manchanda et al. [6]	2012	New Delhi, India	165	0-28 days	35.15%
Sowande et al. [17]	2014	Osun State, Nigeria	110	0-28 days	53.60%
Grabski et al. [18]	2015	Uganda	571	0-16 years	12.40%
de Bruin et al. [5]	2015	Utrecht, Netherlands	45,182	0-18 years	0.13%
Raina et al. [19]	2015	Jabalpur, India	124	0-28 days	30%
Davies et al. [20]	2016	Congo, South Africa, South Sudan	14,482	0-14 years	19.70%
Verma et al. [21]	2016	Rohtak, India	298	0-1 month	16.40%
Talabi et al. [22]	2018	Nigeria	4,108	0-15 years	15.6/10,000
Bonasso et al. [4]	2018	USA	103,444	0-21 years	0.7%
Firomsa et al. [2]	2018	Ethiopia	210	7 days-12 years	4.28%
Ali et al. [23]	2018	Srinagar, India	120	0-28 days	23.33%
Cheung et al. [24]	2019	Uganda	2,090	0-12 years	27.7%
Yassegoungbe et al. [25]	2020	Northern Benin	68	0-12 years	8.80%
Harunani et al. [26]	2020	Kenya	140	0-17 years	20%
Ullrich et al. [27]	2021	Uganda	357	0-14 years	14%
Grabski et al. [18]	2021	Uganda	1,964	0-13 years	2.40%
Newton et al. [3]	2021	Kenya	6,005	0-18 years	1.70%
Didier et al. [28]	2021	Niger	327	0-5 years	5.5%
Tarekegn et al. [29]	2021	Ethiopia	849, 445	0-18 years	2.58%

Discussion

Burden of Disease

Surgical conditions account for 10%-33% of the total pediatric admissions, but emergency situations account for 50%-78% of surgical cases in the pediatric age group [20]. Surgical acute abdominal pain occurred in 10%-30% of children who arrive at the emergency room with acute abdominal pain; however, surgical acute abdominal pain occurred in only 2% of the pediatric patients who came with pain in the abdomen [21]. The most common reason for children's emergency hospitalizations is pediatric abdominal surgical emergency (PASE), which forms approximately 2.4%-3.1% of all admissions in the pediatric age group [22]. About 4% of pediatric admissions in Tanzania are abdominal surgical emergencies according to Mhando et al. [9] (2008). The most common congenital gastrointestinal system defect, Meckel's diverticulum, affected 2% of the population. These diseases are commonly congenital during the newborn period, but with older age, acquired conditions become more common [2]. For intussusception, the commonest age is 3-5 years, with 60% of cases occurring at the age of one year and a peak frequency between the ages of six and 11 months [7]. In the nontraumatic category, incarcerated inguinal hernia (14/31, 45.1%) is the most common cause in newborns, followed by intussusception (13/31, 41.9%) and acute appendicitis in children older than one year (68.7%) [16]. Most of the patients with PASE come with complaints of pain in the abdomen. In 2008, the Centers for Disease Control and Prevention (CDC) reported that abdominal pain accounted for 11% of emergency room visits, and 20% of children who arrived with stomach problems required surgery [24]. On the other hand, neonatal surgical emergencies account for 12.3% of all pediatric surgical admissions [25]. In Uganda, a surgical illness burden of 990 unique presentations and 550 procedures per year was discovered, but only 3.5% of neonatal surgical need was addressed, signifying a significant concealed mortality. In community surveys, more recent LMIC estimates revealed a low surgical volume and significant unmet demand [26]. In comparison to overall emergency surgical admissions, Didier et al. discovered a 4.17% risk of PASE in children under the age of five [28]. Other African authors discovered a rate of 10.32% in children under the age of five [27].

Mortality Associated With Pediatric Abdominal Surgical Emergencies (PASE)

The perioperative mortality rate (POMR) is a metric for determining the quality and safety of surgical care. POMR is the number of all-cause fatalities before discharge in patients who have had an operating room operation divided by the total number of procedures and is given as a percentage [4]. It is a dependable quality and safety indicator for perioperative treatment. Mortality rates range from less than 3% in some etiologies to more than 50% in neonatal intestinal obstruction emergencies [29]. Wound infection, wound dehiscence, anastomotic leaks, intra-abdominal collection, prolonged ileus, adhesive intestinal obstruction, incisional hernia, respiratory tract infection, multiple organ failure, and death are among the postoperative complications following acute abdomen. The majority of these complications are in cases with extensive peritoneal soilage, bowel strangulation, and severe malnutrition. Remote organ failure, postoperative anastomotic leaking, intraperitoneal abscess, and typhoid perforation of the gastrointestinal tract have all been linked to a poor prognosis [24]. Patients who develop complications are more likely to be admitted for an extended period of time, which results in higher hospital costs [22].

Perioperative Mortality Rate (POMR) in High-Income Countries (HICs) Versus Low- and Middle-Income Countries (LMICs)

While the POMR in PASE in the United States has been steadily declining for the past 30 years, preventable morbidity and mortality in low- and middle-income countries (LMICs) has remained high [22]. Only a few case series on pediatric POMR in LMICs have been published in peer-reviewed journals, the majority of which are retrospective and reveal a high mortality rate in children. According to Talabi et al., POMRs within 24 hours, seven days, and 30 days were 113.2, 207.6, and 320.8, respectively, per 10,000 operations [22]. These figures are greater than those reported by HICs. According to a study by the ‪GlobalSurg Collaborative, LMICs have 4-7 times greater 30-day mortality than HICs. In the same study, the 24-hour mortality rates for LICs, MICs, and HICs were 2.6%, 0.7%, and 0.3%, respectively, whereas the 30-day mortality rates were 8.3%, 2.9%, and 0.9%, respectively [30]. Death rates in LMICs are 100 times higher than in HICs, according to Newton et al. [3]. Bonasso et al., from the United States, found a significantly lower 30-day mortality rate of 0.7% [4]. Cheung et al., in a study of 2,090 children, found an overall POMR of 10%, and 27.7% in those with congenital anomalies, while the postoperative POMR was 15% [24].

Late presentation, sepsis, insufficient health personnel, higher American Society of Anesthesiologists Physical Status (ASA PS) III scores, emergency and multiple surgeries, nonadherence to the surgical checklist, advanced diseases, grossly inadequate physiological monitoring facilities, and a lack of a dedicated neonatal intensive care unit (ICU) are the determinants. Emergency operative procedures had a higher POMR than elective procedures in both HICs and LMICs. This is due to the serious condition in which some children with PASE present to the hospital [30]. Anesthesia-related deaths were 2-3 times higher in middle-income nations and could be 1,000-fold higher in some resource-poor countries [22].

Morbidity From Pediatric Abdominal Surgical Emergencies (PASE)

Pediatric abdominal surgery emergency (PASE)-associated perioperative morbidity and death is a major public health concern [13]. Wound infection, wound dehiscence, anastomotic leakage, intra-abdominal collection, delayed ileus, adhesive intestinal obstruction, incisional hernia, respiratory tract infection, multiple organ failure, and death are all possible complications after an acute abdomen. The majority of these issues occur as a result of peritoneal soiling, bowel strangulation, or severe malnutrition. These are frequently caused by a patient's late arrival at the hospital [7]. Remote organ failure, postoperative anastomotic leaking, nonlocalization of an abscess within the peritoneal cavity, and gastrointestinal perforation due to typhoid enteritis have all been linked to a poor prognosis. Patients who develop these complications spend more time in the hospital, which results in increased hospital expenditures [16].

Age and Pediatric Abdominal Surgical Emergencies (PASE)

The age of the pediatric population has been characterized in a variety of ways by various organizations. The American Academy of Pediatrics released a statement in 1988 setting a 21-year upper age limit for the pediatric population, which was reaffirmed in 2012. The United States Department of Health and the Food and Drug Administration utilize the following classification system: neonates, from conception to the age of 28 days; infants, ranging in age from 29 days to less than two years; children, ranging in age from two to less than 12 years; and adolescents, those between the ages of 12 and 21 (up to but not including the 22nd birthday). In the United Kingdom, however, the top age limit is 18 years, while pediatric surgeons in India treat children up to 14 years old [3]. Morbidity is also influenced by one's age. According to the literature, neonates are more prone to perioperative death than older children. Neonates have a 20-fold higher chance of death than their older counterparts [8]. The type of disease, presentation, and type of operation performed also vary according to age. Higher mortality is linked to neonatal age, a 72-hour gap between admission and operation, and postoperative problems [21].

Neonates have unique hurdles as they make the transition from fetal to postnatal life. Infection susceptibility, hypocalcemia, hypoglycemia, and a lack of cardiovascular reserve are all factors to consider (as a result, they cannot tolerate blood loss, as well as adults or older children). They are also prone to extravascular fluid retention, which makes wound healing a difficult metabolic task. As a result, the shock of surgery throws off the delicate balance, putting them at risk for sepsis, anemia, nutritional deficiencies, and respiratory problems [19]. Technologically enhanced neonatal critical care centers, full parenteral nutrition, and extracorporeal membrane oxygenation have mostly addressed these issues in affluent countries. Sepsis, multiple procedures causing repeated exposure to anesthetic agents in the newborn period, blood loss and the intricacy of the congenital defect, delayed presentation, lack of skilled staff, and financial issues on the part of the caretakers are all etiological variables [3]. Congenital deformities have a higher mortality rate than acquired illnesses. Prematurity appears to affect the chances of survival in several congenital conditions. Gestational age is one of the six preoperative risk factors for postoperative morbidity and death [19]. Cardiovascular comorbidities and ASA PS > 3 (III) are also factors to consider. According to Akbilgic et al., infants born prematurely have a fourfold increased risk, whereas neonates undergoing surgery have a 20-fold increased risk of sudden mortality within 30 days of surgery than older children [22]. In impoverished nations with limited resources, newborn surgery is still a high-risk procedure with a high death rate. As a result, it is recommended that neonates be operated on only by pediatric surgeons and that proper resuscitation and diligent monitoring be undertaken to enhance surgical results [3].

In a case series of 334 children, Ghritlaharey et al. found that 10.17% died; 7.18% were males and 2.99% were females, and 2.69% were infants, 3.29% were children from one to five years of age, and 4.19% were children from five to 10 years of age [14]. Similarly, a 10.1% mortality rate has been reported by Ademuyiwa et al., of which 25.6% were neonates and 2.3% of 86 cases were in all other age groups (P = 0.002) [15]. Death within 72 hours of surgery was seen in 6.5%, and 22.7% died after 72 hours of surgery (P = 0.003). Abantanga et al. from Ghana found a similar POMR of 9.7% [12], and Mhando et al. from Tanzania reported a higher POMR of 34% [9]. Talabi et al. found that neonatal fatalities account for 58.8% of all deaths. The significant fatality rate in this age group of 2,439 per 10,000 procedures within 30 days necessitated a review of newborn surgical programs. The major causes of death in these infants were being very sick at admission and gastroschisis, necrotizing enterocolitis, and esophageal atresia. When compared to acquired disorders, congenital anomalies were linked to a 12.7-fold increased risk of death in neonates [22].

Males were shown to have a twofold higher risk of death than females when they were diagnosed with PASE [8]. However, in some studies, gender did not play a major role in perioperative deaths [29].

Etiological Factors Related to Pediatric Abdominal Surgical Emergencies

Esophageal atresia (40%-90%) is the cause of the highest mortality rate in the neonatal age group, followed by midgut volvulus (22%-45%), jejunoileal atresia (14%-56%), congenital diaphragmatic hernia (n = 3/7, 42%), and necrotizing enterocolitis (n = 11/35, 31%). Duodenal atresia (0%-40%), typhoid intestinal perforation (9%-28%), corrosive esophageal injuries (2%-25%), anorectal abnormalities (0%-25%), Hirschsprung's disease (0%-23%), and spina bifida (6%-14%) all have lower POMR [26]. The case fatality for typhoid perforation alone is around 12.6% [22]. When compared to other indications, perforation in visceral organs is substantially related to greater 30-day mortality; acute appendicitis, on the other hand, is linked to lower 24-hour and 30-day mortality [30]. Patients with typhoid ileal perforation have high mortality rates, with 15.8% overall mortality and 14.4% postoperative mortality. Other markers include etiological variables such as sepsis, multiple anesthetic exposures in the newborn period, and surgical hemorrhage, as well as the intricacy of congenital defects, delayed presentation, lack of skilled staff, and budgetary constraints on the caretakers' part [16]. The presence of sepsis at the time of admission is a statistically significant predictor of mortality. Poor transportation, late presentation at the hospital, insufficient equipment and facilities, insufficient staff, inadequate documentation, and poor follow-up are among the other problems. Furthermore, many infants die before ever reaching a clinic that can perform neonatal surgery, implying that there is additional "hidden" mortality [2].

Ekenze et al. from Nigeria, in a retrospective analysis of 130 pediatric patients with intestinal obstruction, found an overall POMR of 3% [13]. Bowel obstruction was caused by a congenital inguinal and umbilical hernia, duodenal/jejunoileal atresia, Hirschsprung's disease, and anorectal malformations, as well as acquired disorders such as hypertrophic pyloric stenosis, intussusception, adhesions, and worm impaction [30]. A sorry tale of 10 infants was reported from Kenya who had esophageal atresia; two were brought to the hospital after six days of life with severe malnutrition, sepsis, and pneumonia. A gastrostomy tube replacement was performed for supplemental nutrition; however, they could not be saved. For two others who reported after six days of life, definitive treatment was performed, but they died in the early operative period. The other six reported prior to day 3 of life and could be saved [26].

Access to Care and Perioperative Mortality in PASE

Access to care has been a major determinant in various countries, and distance from a care facility is a bottleneck, especially in low-resource settings. The World Health Organization advises that surgical treatment centers be provided within 50 km of residence. Cheung et al. reported a travel distance ranging from 0 to 1,000 km, with a median of 30 km. In their study, 11 children traveled between 500 and 1,000 km. Cost, transportation, referral system, and nonavailability of specialists all are responsible for access away from the local area as no pediatric surgeon may be available. Median admission to surgery interval is obviously a factor that impacts surgery outcomes. Delay may be due to hospital systems, availability of blood and components, retrieving diagnostic results, economies, policies of self-funding, and healthcare costs [24]. Ademuyiwa et al. reported a median admission to surgery interval of 52 hours, which may prove detrimental [15]. Untoward consequences are seen in Nigeria, where parents cater to healthcare costs of children/wards, and it takes days to raise funds [17]. In pediatric surgical emergencies, a delay in intervention of more than 72 hours, neonatal age, and severe postoperative sequelae are all linked to a greater fatality rate [28]. Ali et al. from Kashmir, India, found that delay in presentation of >2 days after the onset of symptoms was a significant risk factor for mortality [23].

Late presentation was attributed to poor recognition of illness due to the following: a) ignorance and lack of awareness, b) delay in seeking and access to care due to financial constraints, c) lack of adequate means of transportation, d) poor parental motivation, and e) delay in referral from primary first contact physicians due to delay in diagnosis [23]. Other factors mitigating surgical safety are constraints of infrastructure, manpower crunch, dearth of pediatric intensive care units, faulty health insurance schemes, and deficient monitoring equipment [3].

Electrolyte Imbalance in Pediatric Abdominal Surgical Emergencies

Electrolyte imbalances are prevalent in young children and can cause delays in management. Deranged kidney function tests, particularly serum potassium, have been linked to mortality in patients with PASE [26]. Thrombocytopenia, acute kidney injury, metabolic acidosis, and coagulopathy on admission are all significant predictors of mortality [6].

Blood Transfusion

The role of transfusion as an independent morbimortality factor has been established in pediatric surgery and critically unwell pediatric patients. Transfusion is an independent risk factor for perioperative complications, repeat surgery, increased duration of ICU admissions, and risk of morbidity and mortality [10]. Patients who need transfusion have more comorbidities and are exposed to hemorrhagic surgery. Optimized transfusion techniques could improve patient outcomes. Restrictive transfusion procedures and using viscoelastic methods at the bedside can decrease exposure to blood products [10].

Continued Postoperative Mechanical Ventilation

Continued postoperative mechanical ventilation after surgery is a significant risk factor for mortality. The reason for the bad prognosis in such patients who need continued postoperative mechanical ventilation is the bad general condition prior to surgery [17]. Patients who are too sick are more likely to need postoperative mechanical ventilation. The second reason is the complications associated with mechanical ventilation. Thus, every effort should be made to make patients breathe spontaneously without ventilatory support postoperatively by proper preoperative optimization, avoiding unnecessary delay in surgery, dedicated anesthetic care including warm care during surgery, and decreasing operative time by doing the minimum that is necessary for sick neonates [21].

Indicators of Survival

The idea behind early admission to the hospital as a factor in reducing morbidity and mortality in children is that early surgical intervention is possible. A variety of parameters must be met for good surgical results, including skilled staff, proper surgical facilities and supplies, and timely surgical care [3]. As a result, any single intervention in this complex system is likely to fall short of properly addressing these challenges [18]. Temporary platforms such as "surgical safaris" or short-term training courses outside of one's typical job setting are unlikely to have a long-term influence [12]. Long-term institutional cooperation is the most likely situation in which broad systemic changes can occur [20]. The increased global recognition of pediatric surgery as a crucial component of healthcare has provided a one-of-a-kind incentive for the provision of much-needed surgical services, particularly in low- and middle-income nations. The challenge ahead in terms of access to and quality of care is huge [11]. In the care of acute abdomen in children, nothing can substitute a physician's clinical acumen; thus, training is important. The availability of computed tomography (CT) scans, magnetic resonance imaging (MRI), and other scanning and laparoscopic treatments plays a critical role in the survival of children in the present era of technology [10].

Because many pediatric surgical situations are urgent, timely access to care is critical. Increased distance from healthcare facilities causes patient delays, as well as higher rates of morbidity and mortality [22]. Surgery performed within 72 hours is linked to a higher rate of survival than surgery performed after a longer period of time. While attempts should be made to decrease the time between admission and surgery, the dearth of pediatric critical care facilities precludes preoperative resuscitation affecting surgical results [17]. Proposed cost limits can be a disincentive, as can a lack of insurance options. To have a favorable impact on reducing admission to surgery, intervention time, and eventual survival rates, hospitals should be encouraged to waive surgical and diagnostic charges in PASE through national policy.

## Conclusions

Surgical emergencies account for more than half of all surgical admissions and a significant portion of surgeons' workload in most parts of the world. Pediatric abdominal surgical emergencies encompass a wide range of diseases, ranging from congenital to acquired disorders. When compared to elective surgeries, they are associated with higher morbidity and death, particularly in underdeveloped nations. Neonatal age, late presentation to a healthcare facility, delay in diagnosis and treatment, and postoperative morbidity are all factors that contribute to increased mortality in resource-poor countries. Prompt diagnosis, resuscitation upon arrival, decreased surgery intervention time, expert operating care, and postoperative management in a dedicated critical care unit are all essential for survival.
